# **Scalable assembly of** ***Ascaris*** **mitogenomes from whole-genome data reveals a novel clade**

**DOI:** 10.1038/s41598-026-65562-w

**Published:** 2026-09-11

**Authors:** Lauren Woolfe, Kezia Kozel, Poom Adisakwattana, Allen Jethro Alonte, Kennesa Klariz Llanes, Alexandra Juhász, J. Russell Stothard, Christina Strube, Marie-Kristin Raulf, Scott P. Lawton, Toby Landeryou, Vachel Gay Paller, Umer Chaudhry, Arnoud H. M. van Vliet, Martha Betson¹

**Affiliations:** 1 Discipline of Comparative Biomedical Sciences, School of Veterinary Medicine, University of Surrey, Guildford, UK; 2 Department of Helminthology, Faculty of Tropical Medicine, Mahidol University, Nakhon Pathom, Thailand; 3 Institute of Biological Sciences, College of Arts and Sciences, University of the Philippines Los Baños, Laguna, Philippines; 4 Department of Tropical Disease Biology, Liverpool School of Tropical Medicine, Liverpool, UK; 5 Institute of Medical Microbiology, Semmelweis University, Budapest, Hungary; 6 Institute for Parasitology, Centre for Infection Medicine, University of Veterinary Medicine Hannover, Hannover, Germany; 7 Centre for Epidemiology and Planetary Health, School of Veterinary Medicine & Biosciences, Scotland’s Rural College, Inverness, Scotland, UK; 8 Department of Veterinary Biomedical Sciences, Lewyt College of Veterinary Medicine, Long island University, New York, USA

**Keywords:** Soil-transmitted helminths, *Ascaris lumbricoides*, *Ascaris suum*, Genome assembly, Mitochondrial genome, Parasite genomics, Computational biology and bioinformatics, Evolution, Genetics

## Abstract

The genus *Ascaris* is an important group of giant parasitic roundworms, infecting over 700 million people globally and causing substantial economic losses in domestic pigs. Whilst species of *Ascaris* are morphologically indistinguishable, analysis of mitochondrial loci has revealed three clades (A, B, C) broadly associated with host species and geographic distribution. The diversity within these lineages may expand with the addition of further genomic data. Here, we present a bioinformatic framework for *de novo* assembly of complete mitochondrial genomes (mitogenomes) from low-coverage whole-genome data through host-read depletion or mtDNA read enrichment, followed by mtDNA-specific assembly. Our approach yielded 149 high-quality *Ascaris* mitogenome assemblies, enabling the study of population-level diversity, including the identification of a novel clade (Clade D, designated here) associated with human samples from Ethiopia. Our analysis further revealed Clade C to comprise of pig-derived samples from Europe based on characterisation of worms isolated in Germany. The methods described here provide a scalable framework for mitogenome reconstruction with insights into roundworm population-genomic and phylogenetic studies.

**Supplementary Information:**

The online version contains supplementary material available at https://doi.org/10.1038/s41598-026-65562-w.

## Introduction

Nematodes are a diverse group of worms capable of inhabiting a wide variety of ecological niches, including soil, fresh and saltwater ecosystems, and parasitise a broad range of plants and animals, including humans^1–3^. Parasitic nematodes are responsible for substantial morbidity and mortality in affected populations and have global economic implications, particularly in agriculture and healthcare^1,4–6^. Recognising their significant public health impact, the World Health Organisation has specifically targeted soil-transmitted helminths (STHs)—a subgroup of nematodes comprising genera such as *Ascaris*, *Trichuris*, *Ancylostoma* and *Necator* —for elimination as a public health problem by 2030^7^. Concurrently, veterinary medicine faces increasing challenges from helminth infections, especially in livestock and companion animals, where the rise of anthelmintic resistance is resulting in reduced treatment efficacy, compromised animal welfare, decreased productivity, and economic losses^8–10^.

The most prevalent STH in humans is the giant roundworm *Ascaris*, which once mature can reach up to 30 cm in length and resides in the small intestine of the infected host. Spread through the ingestion of eggs defaecated by an infected host, *Ascar*is is suspected to infect over 700 million people worldwide^11,12^ and is considered one of the most prevalent helminths in domestic pigs^13^. Historically, *Ascaris* worms from pigs have been classified as *A. suum* while those from humans have been designated as *A. lumbricoides*. However, more recent evidence has demonstrated that this distinction is not strictly defined by host. These two species are morphologically indistinguishable, often capable of cross-transmission and can hybridise with evidence of genetic introgression. Consequently, their taxonomic relationship—whether they represent distinct species or a single shared species—remains under debate^14–16^.

Genomic markers provide powerful tools for detecting genetic variation, reconstructing evolutionary relationships, and monitoring phenotypic traits such as drug resistance or adaptation to environmental stressors^17–20^. In recent years, mitochondrial genomes (mitogenomes) have emerged as highly valuable genetic markers across diverse taxa due to their compact size, elevated copy number relative to nuclear DNA and conserved gene content^21,22^. Typically, nematode mitogenomes are comprised of a single circular genome encoding 12 protein-coding genes, 2 ribosomal RNA (rRNA) genes, and 22 transfer RNA (tRNA) genes., where gene size and organisation are generally well conserved across species^23^. Owing to its lack of recombination and maternal inheritance^24–27^ and high degree of genomic plasticity^23^, mtDNA markers have become increasingly important in DNA barcoding (using standardized gene fragments, such as cytochrome oxidase subunit 1 [*cox1*] and NADH dehydrogenase subunit 1 [*nad1*], to distinguish between closely related lineages or genotypes), evolutionary studies, and species identification^2^.

First described in 1992^28^, the *Ascaris* mitochondrial genome has been well studied, enhancing our understanding of *Ascaris* population structure through the use of targeted individual markers. Among the most frequently used markers is the gene encoding *cox1* – a mitochondrial protein integral to oxidative phosphorylation. Its widespread use largely reflects the availability of extensive comparative datasets rather than its suitability as the most informative marker, as *cox1* is among the more conserved mitochondrial genes^29,30^. Nevertheless, *cox1* analysis has led to the identification of over 70 distinct haplotypes that cluster into three distinct clades (A-C), which do not clearly segregate by host^31^. Clade A incorporates isolates from pigs in non-endemic regions and humans in endemic regions, while clade B includes mixed isolates from both pigs and humans which are geographically widespread irrespective of endemicity. In contrast, clade C predominantly comprises pig-derived isolates from Europe, and small number of human-derived isolates from non-endemic regions indicating potential zoonotic transmission^31^. This largely pig-associated clade forms a distinct genetic cluster^29,31–33^ and is suggested to have diverged as recently as 2500 years ago^33–36^. Comparatively, clade A and B are suspected to have split more recently, as early as 300 years ago, and have been readily dispersed through international movement of people and pigs^34^.

While *Ascaris* remains one of the most prevalent helminth infections globally, key aspects of its population biology and transmission dynamics are still poorly understood^16^. The close genetic similarity between *A. lumbricoides* and *A. suum* (overall genomic divergence is < 2%)^29^, coupled with evidence of hybridisation and introgression in regions where humans and pigs coexist, continues to obscure species boundaries and complicate the assessment of zoonotic potential^31,37,38^. Compared with many other nematodes, *Ascaris* nuclear genomes are notably large, ranging from 270 to 300 Mbp and containing approximately 18,000 protein-coding genes^39^. While whole-genome approaches are essential for resolving fine-scale processes such as introgression and recombination, their application at population scale remains challenging, particularly in resource-limited settings. Population-level molecular studies have been further limited by the cost and complexity of sequencing and by reliance on single-gene mitochondrial markers (e.g. *cox1*), which capture only a subset of mitochondrial variation and may provide insufficient resolution to infer population structure, gene flow, or the sources of reinfection following treatment. As a result, in-depth genomic data from endemic regions remain scarce, and the relative contribution of human and animal reservoirs to ongoing transmission is not well defined. To achieve the WHO 2030 target, more detailed understanding of parasite population structure and transmission in endemic settings is essential to guide future diagnostic and control strategies.

While single-marker and reference-guided approaches (i.e. mapping reads to a reference genome via alignment and variant calling, or to isolate mtDNA reads prior to assembly) have provided valuable insights into nematode population structure, they can be limited in resolution, particularly when distinguishing closely related lineages or working with complex, low-parasite DNA samples. *De novo* mitochondrial genome assembly from whole-genome sequencing data therefore provides a practical complementary approach. Genome-skimming workflows can recover high-copy mitochondrial genomes from low-depth sequencing data, offering greater phylogenetic resolution than single-gene markers and supporting scalable analysis across multiple samples. In helminths, genome skimming has been shown to identify single- and multi-species infections and, in some samples, recover sufficient mitochondrial coverage for phylogenetic analysis^40,41^. However, broader implementation in endemic settings remains dependent on sample quality, parasite DNA abundance, sequencing infrastructure, computational capacity, and robust reference datasets that capture the geographic diversity of circulating *Ascaris* populations, particularly given evidence that population-biased variation in soil-transmitted helminths can affect molecular diagnostic targets^40,42^.

Complete mitochondrial genomes can provide greater resolution of *Ascaris* mitochondrial diversity than commonly used single-gene markers. However, the systematic recovery of complete mitogenomes from existing low-coverage whole-genome sequencing datasets are not yet commonly applied to STHs. Here, we use publicly available, previously validated bioinformatic tools within a consistent and scalable workflow to reconstruct complete mitogenomes, evaluate the effects of host-read depletion and mitochondrial DNA enrichment on mitogenome recovery, and characterise how mitochondrial diversity is distributed across individual loci, including the extent to which commonly used markers such as *cox1* reflect the variation captured by the complete mitochondrial genome. We then apply this approach to 149 heterogeneous *Ascaris* whole-genome datasets to investigate geographical and host-associated patterns of mitochondrial genetic clustering.

## Methods

### Sample information and initial processing

Twenty-four *Ascaris* worms obtained from humans and pigs from different countries worldwide were included in this study. Seven adult *Ascaris* samples from Germany were collected from slaughtered pigs at an abattoir located in the federal state of North Rhine-Westphalia (samples DE_P6-P24). Additionally, one adult worm was sent as diagnostic material to the Institute for Parasitology, University of Veterinary Medicine Hannover, from a human patient from the federal state of Schleswig-Holstein suffering from recurrent *Ascaris* spp. infection with suspected zoonotic transmission due to backyard pig farming (sample DE_H1a). Information on the origins of other samples and downloaded mitogenomes is summarised in Supplementary Table S1. Ethical approvals for the original sample collections were obtained by the respective research groups and granted by the relevant institutional review boards and national authorities, as reported in the corresponding publications (see Table S1 for full details). All original studies were conducted in accordance with the appropriate institutional, national, and international guidelines and regulations.

In addition, Illumina sequencing reads from 125 *Ascaris* worms were downloaded from the NCBI Sequence Read Archive (https://www.ncbi.nlm.nih.gov/sra), while an additional 18 *Ascaris* mitogenomes and 1 *Toxascaris* mitogenome were obtained from NCBI Nucleotide (https://www.ncbi.nlm.nih.gov/nucleotide). This study did not involve the collection of new biological samples; rather, it analysed previously collected samples and publicly available sequencing data.

### DNA extraction

DNA was extracted from ~ 1-inch sections of worm tissue. To minimise contamination from fertilised eggs and male sperm (which could lead to spurious genotypes^43^), the uteruses and intestines were removed. Muscle tissue was separated from the cuticle and processed in-house using the Qiagen DNeasy Blood & Tissue Kit (Qiagen, Hilden, Germany; Cat. No. 69506) or the MasterPure Complete DNA and RNA Purification Kit (Lucigen, Middleton, WI, USA; Cat. No. MC85200) following the manufacturers’ protocols. For the Qiagen kit, DNA was eluted in 50 µl of AE buffer, which was re-applied to the spin column to maximise DNA concentration.

DNA concentration and purity were assessed using the Invitrogen Qubit™ 1X dsDNA High Sensitivity quantification kit and NanoDrop spectrophotometer, respectively. Only samples with a concentration ≥ 5 ng/µl and A260/A280 ratio ≥ 1.6 were utilised for sequencing. Whole-genome sequencing was performed by Novogene Europe (Cambridge, UK) using the Illumina NovaSeq X Plus platform with 150 bp paired-end reads, at 30X coverage.

### Processing genomic data

#### Read processing and quality control

For all samples, raw sequence reads were processed with FastP (v0.23.4)^44^ to remove any remaining adapter sequences and low-quality reads. Seqtk (version 1.5-r133)^45^ was employed to subsample each paired-end read set to 20 million (20 M) reads, totalling 40 M reads per sample. Those samples which had less than 20 M reads each way were not subsampled but still utilised in the analysis. Quality control of the subsampled reads was assessed using FastQC (version v0.12.1)^46^.

#### Host depletion and mitochondrial read enrichment

We compared whether host depletion or mtDNA enrichment improved the assemblies using Bowtie2 (Version 2.5.4)^47^(host depletion only) and Deacon (Version v0.7.0)^48,49^. For Bowtie2, a host reference database was constructed using Bowtie2-Build, incorporating the pig (GCF_000003025.6) and human (GCF_000001405.40) reference genomes, including their mitochondrial sequences. Subsampled 2 × 20 million paired-end reads were aligned to deplete any reads mapping to the host genomes^50^ using the SQM_mapper script version 1.65post1^51^. A similar process was carried out for Deacon whereby a minimizer index file was created using the aforementioned human and pig reference genomes for depletion of host genome reads^48,49^. For mtDNA enrichment using Deacon, reads were filtered against a k-mer database of *Ascaris* mitochondrial genomes, and only matching reads were retained for genome assembly. This database was constructed from a range of ascarid mitogenomes (see Supplementary Material for details).

#### *De novo* mitochondrial genome assembly

**Fig. 1:**
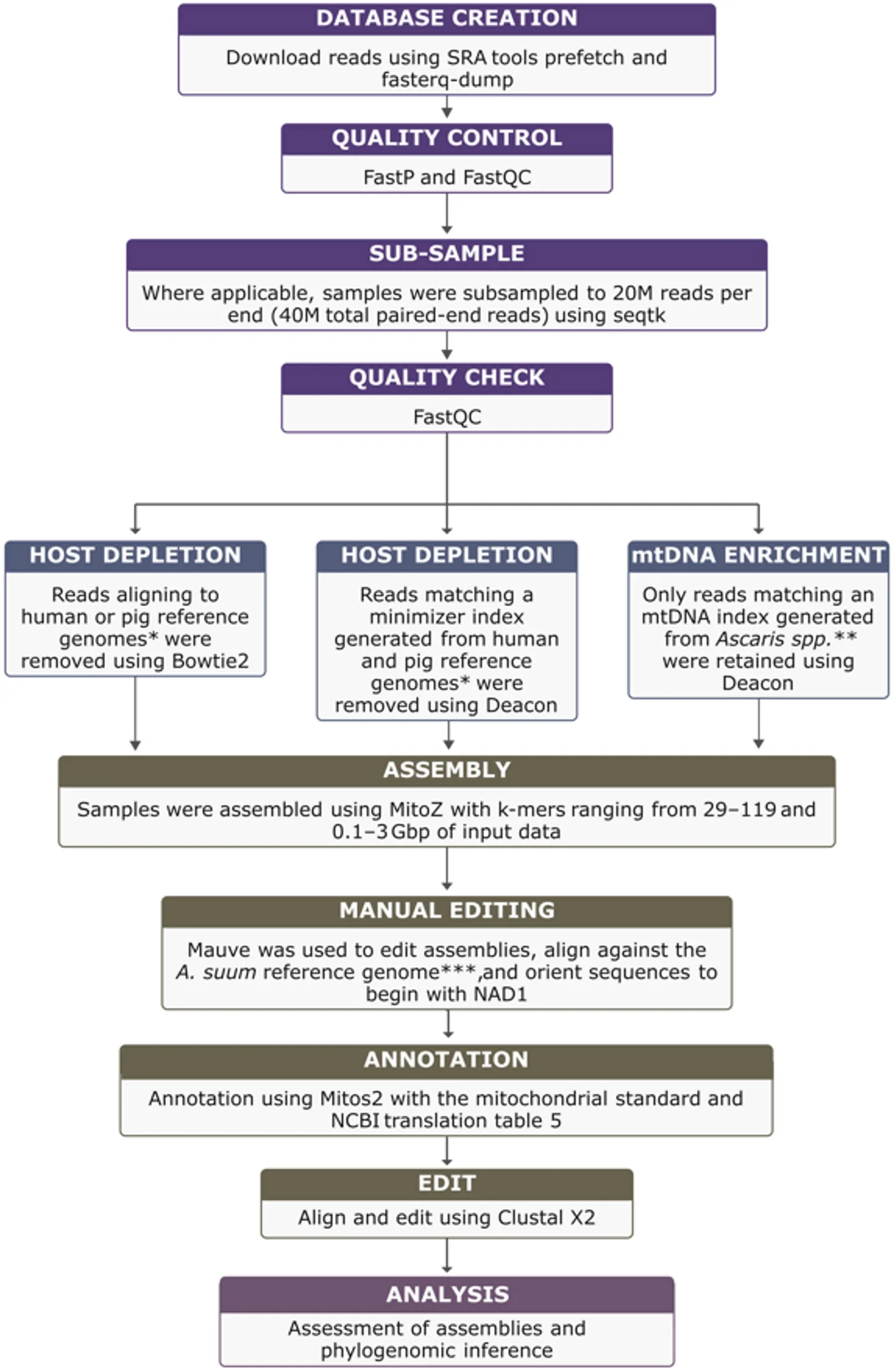
de novo assembly and analysis of mitochondrial genomes from raw sequencing data. Raw reads were downloaded and quality-controlled, followed by subsampling and secondary quality assessment. Reads were then processed using either host depletion (Bowtie2 or Deacon) or mitochondrial enrichment (Deacon) approaches prior to assembly with MitoZ. Resulting assemblies were manually curated, annotated, and aligned before downstream phylogenomic analyses. *Host reference genomes used for depletion were human (GCF_000001405.40) and pig (GCF_000003025.6). **The mitochondrial reference database used for enrichment comprised a range of ascarid mitogenomes (see Supplementary Material for full accession list). ***Reference genome used for alignment and orientation was GCA_013433145.1.

*De novo* assembly of mitogenomes was performed using MitoZ (Version v3.6)^52^, initially employing *k*- of 39, 59, 79, 99, and 119 for all samples, except for those from Kenya^29^, which had an average sequencing read length of 100 bp. Therefore, the *k-* of 119 was removed and the *k-* of 29, was added. Initially, 1 Gbp coverage was used for raw reads post-decontamination using Bowtie2 or Deacon, whereas 0.1 Gbp was utilised for the mtDNA alone. For those that failed assembly or produced partial or very large assemblies, further amendments to the *k* values (addition of k = 21 or k = 89) and lowered coverage requirements were used to achieve complete genome assembly. Sequence length and N_50_ for each assembly was assessed using QUAST (version 5.02)^53^. The workflow is depicted in Fig. 1.

#### Assembly validation and quality assessment

Each resulting FASTA file was then visually inspected using Mauve Aligner (version 2015-02-26)^54^ to multi-align to the reference genome. When required, manual editing was performed to transform sequences into reverse complement and to linearise/align all sequences to start at the repetitive 5’ region ahead of the *nad1* gene. Annotation was performed using the standalone version of MitoS2 (version 2.1.9)^55^ using the mitochondrial standard and NCBI translation Table 5 (invertebrate mitochondrial code). Clustal X2^56^ was used to align and edit the resulting annotations.

Thirty samples—including all samples which were sequenced in this study and a subset of samples where whole genome sequence data was previously published (SRR31675200, SRR31675201, SRR31675202, SRR8419293, SRR8419294 and SRR8419295) — were aligned back to their respective assemblies to confirm that each assembly accurately represented the original sequencing reads. To achieve this, each assembly was indexed as a reference genome using Bowtie2-build, and the corresponding 20 M subsampled reads were aligned back to their assemblies using Bowtie2 with the very-sensitive setting. Samtools (Version 1.22.1)^57^ was used to ascertain coverage and average depth.

#### Genome annotation and dataset compilation

All assemblies were annotated using Mitos2 version 2.1.9 using codon translation Table 5, the standard start/stop predictions and the NCBIcode start/stop predictions, with the NCBIcode prediction used for the *atp6*, *cox1*, *cox2*, *cox3*, *nad2*, *nad3* and *nad6* genes, and the standard start/stop prediction used for the *cob*, *nad1*, *nad4*, *nad4L* and *nad5* genes. For *cox1*, the annotation software missed the first 8 amino acids which were excluded from further analysis. All samples were oriented to begin with the with *nad1*, the repetitive OH region removed and were successfully annotated for all 12 protein-coding genes (Supplementary Figure S1).

We downloaded additional 19 complete mitogenomes from GenBank encompassing both human and porcine hosts (*n* = 13), but also worms from non-human primates (*n* = 2), sheep (*Ascaris ovis*, *n* = 3) and one *Toxascaris leonina* sample (canine, China) to be used as an outgroup; the accession numbers are provided in Supplementary Table S1. This resulted in a final dataset of 168 samples.

### Phylogenomic inference of *Ascaris* spp

For both nucleotide and amino acid analyses, each annotated protein-coding gene was manually extracted into individual files and then concatenated in the order present in the assembly (*nad1–atp6–nad2–cob–cox3–nad4–cox1–cox2–nad3–nad5–nad6–nad4L*), excluding tRNA and rRNA genes, and repetitive OH region.

Neighbour-joining trees with bootstrap support (1,000 replicates) were constructed in MEGA12^58^ for the 12 individual gene alignments (nucleotide only) as well as for the 2 concatenated sequences. A neighbour-joining approach was chosen because it offers a computationally efficient means of visualising relationships among closely related mitogenomes and has been widely applied in previous ascarid phylogenetic studies^32,59^. The resulting phylogenetic trees were visualised and analysed in R (version 4.5.2)^60^, using packages *ape* (v.4.5.2)^61^, *ggtree* (v4.04)^62^, *treeio* (v1.34)^63^ and *ggplot2* (v4.5.2)^64^. A heatmap illustrating clade assignment per gene for each sample, and plots demonstrating relationships among continent, host, and clade were created, also using R (version 4.5.2)^60^, using additional packages *dplyr* (v4.5.2)^65^, *readr* (v2.2)^66^, *tidyverse* (v.2.0)^67^ and *patchwork* (v1.3.2)^68^.

To assess whether the major mitochondrial clusters identified by the neighbour-joining analysis were also recovered using an alternative model-based method, maximum-likelihood phylogenetic inference was performed using IQ-TREE v2, with the best-fit substitution model selected using ModelFinder. Branch support was assessed using 1,000 ultrafast bootstrap replicates and 1,000 SH-aLRT replicates. The resulting tree was visualised and annotated in the Interactive Tree of Life (iTOL) and rooted using *Toxascaris leonina* as an outgroup.

To assess if the resulting the assemblies retain the expected population-level variation, genetic diversity indices were calculated for each coding gene and concatenated dataset using DnaSP (version 5)^69^, using the DNA polymorphism function.

## Results

### *de novo* mtDNA assembly of *Ascaris* spp

We generated Illumina sequence data from 24 *Ascaris* samples from pigs and humans, from varied geographical sources (Germany, Thailand, Philippines) and combined them with downloaded Illumina fastq files from human samples from Kenya^29^ and Ethiopia^70^. The final dataset included 134 samples from humans and 15 from pigs. Geographically, the samples represented three continents: Africa (Ethiopia [*n* = 61], Uganda [*n* = 7], Kenya [*n* = 64]), Asia (Philippines [*n* = 4], Thailand [*n* = 3]), and Europe (Germany [*n* = 8], Hungary [*n* = 1], United Kingdom [*n* = 1]).

Each set of paired end sequencing reads was randomly subsampled to 2 × 20 million reads and assembled with MitoZ either without further processing, depletion of pig and human reads with Bowtie2, depletion of pig and human reads with Deacon and filtering of mtDNA-specific reads with Deacon. Assemblies were measured from the first tRNA upstream of *nad1* (trnN) to the last tRNA downstream of *nad4L* (trnS2). Assemblies were then classified as *complete* when a single contig, which contained all 12 protein coding genes was produced; *partially complete* when full-length mitogenome was recovered, inclusive of the presence of the 12 coding genes but included additional contigs; *incomplete* when contigs were generated but no full mtDNA sequence was obtained; and failed when no assembly could be resolved.

Individually, each method successfully recovered complete mitogenome assemblies for > 95% (*n* = 142–146) of samples. Assembly lengths were highly consistent across methods, producing single contigs of approximately 13.4 kb containing all expected protein-coding genes. The mean length ranged from 13,395 to 13,406 bp, consistent with the average calculated from previously published assemblies (13,381 ± 26 bp; Table 1, Supplementary Table S2). Variance was lowest for Deacon depletion (13,395 ± 19 bp) and mtDNA-enriched assemblies (13,397 ± 30 bp), indicating more consistent structural recovery compared to unfiltered data (13,406 ± 115 bp).

Across bioinformatics methods, the Deacon depletion approach performed best overall, producing the highest number of complete assemblies (98%, *n* = 146) and the fewest incomplete assemblies (~ 1%), with no failed samples. Similarly, Bowtie2 depletion showed comparable performance (97% complete assemblies, *n* = 144), although a greater number of these completed assemblies (*n* = 4) slightly exceeded the expected genome length compared to Deacon depletion (*n* = 1) (Supplementary Table S2). Both unfiltered and mtDNA enriched samples performed worse than the depleting methods, producing ~ 95% complete assemblies (*n* = 142), due to increased incomplete (*n* = 3 and *n* = 4, respectively) and failed assemblies (*n* = 3 and *n* = 2, respectively).

Across all 149 samples, only one sample (SRR31692059) failed to yield a complete mitogenome using any of the four approaches. Although each individual pipeline recovered complete mitogenomes for > 95% of samples (*n* = 142–146), the small number of incomplete (*n* = 2–4) or partially complete assemblies (*n* = 1) differed among methods rather than recurring consistently in the same samples. For instance, SRR31757545 was incomplete only under mtDNA enrichment, while the single partially complete assemblies were observed for SRR31692075 under both depletion methods, BunawanS9 under the unfiltered approach, and SRR31675203 under mtDNA enrichment. Notably, one sample (SRR31692075) was fully resolved in a single contig only with unfiltered data; other approaches either generated multiple contigs (depletion methods) or a single contig with apparent mirrored duplication of the mitogenome (mtDNA enrichment).

**Table 1:** Assembly outcomes across unfiltered, host depletion and mtDNA enrichment approaches. Assemblies were classified as *complete* when a single contig containing all 12 coding genes was produced; *partially complete* when full-length mitogenome was recovered but included additional contigs; *incomplete* when contigs were generated but no full mtDNA sequence was obtained; and *failed* when no assembly could be resolved.

Method	Complete (%)	Partially complete	Incomplete	Failed	Average size trnN-trnS2 (bp)
Unfiltered	142 (95.30%)	1	3	3	13,406 ± 115
Bowtie2 Depletion	144 (96.64%)	1	4	0	13,400 ± 47
Deacon Depletion	146 (97.99%)	1	2	0	13,395 ± 19
Deacon mtDNA enrichment	142 (95.30%)	1	4	2	13,397 ± 30

Assemblies obtained following Bowtie2-based host depletion were designated as the reference approach. For datasets that could not be resolved using this method, the most complete alternative assembly (defined as the presence of all 12 coding genes) was selected for downstream analyses. Only one sample (SRR31692059) did not have a single contig assembly and required manual editing to resolve into a single contig, with a gap of 17 nucleotides based on alignment with other *Ascaris* mtDNA genomes, located in the intergenic region between the *cox1* and *cox2* genes.

To assess assembly quality and representativeness, the original sequencing reads were mapped back to their respective *de novo* mitogenome assemblies (see Supplementary Table S3). Of the 30 samples that underwent mapping, a very high coverage was achieved, with most samples exceeding 10,000×. The mean base depth ranged from 937× to 158,022×, while mean base quality scores ranged from 34.8 to 39.5, and average mapping quality was consistently high (≥ 41.4) across all samples. Importantly, all assemblies achieved > 99.8% genome coverage.

### Identification of a novel mtDNA clade for *Ascaris*

**Fig. 2:**
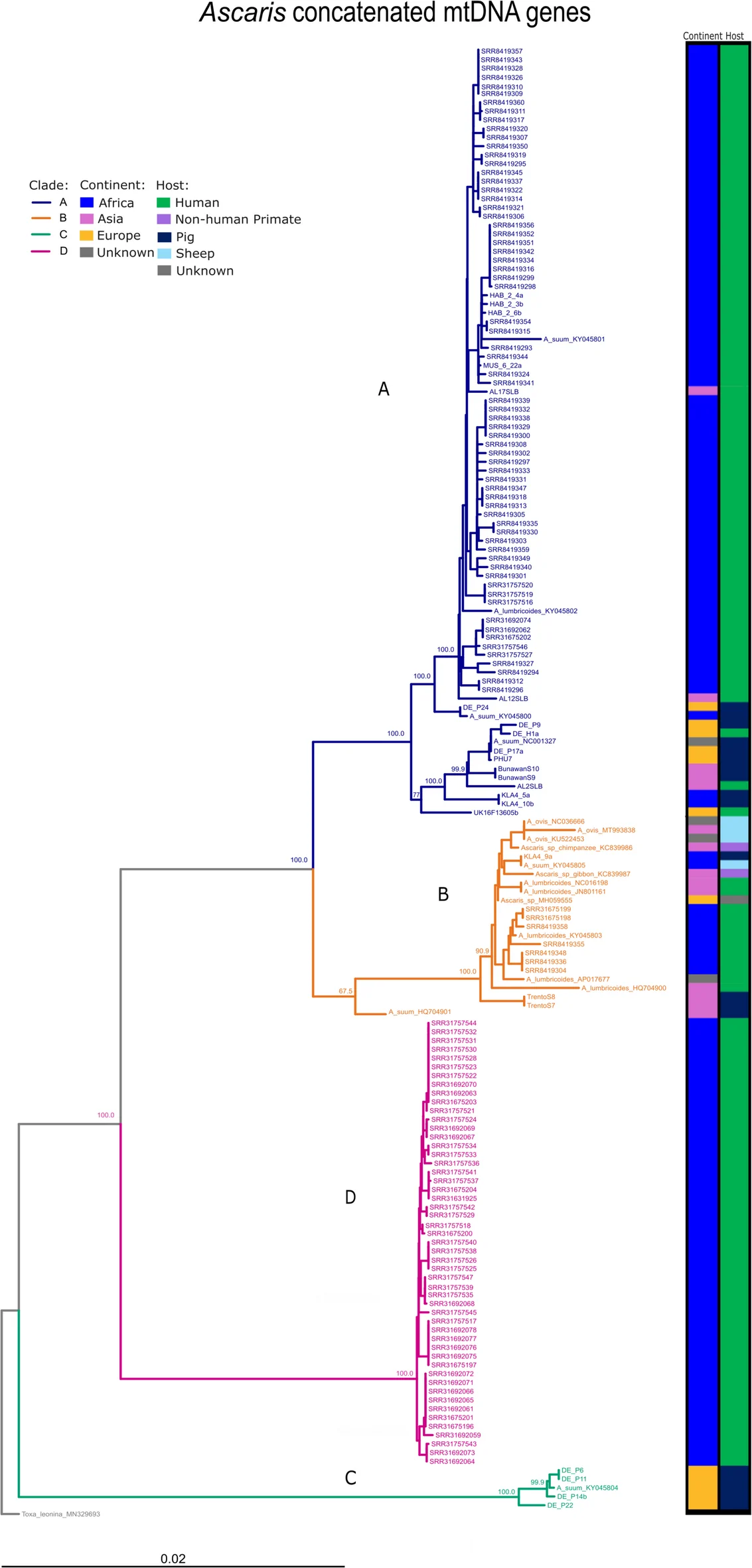
*Neighbour-joining tree of concatenated mitochondrial DNA sequences from* Ascaris *samples.* The tree was constructed using concatenated nucleotide sequences from 12 mitochondrial protein-coding genes in the order: *nad1–atp6–nad2–cob–cox3–nad4–cox1–cox2–nad3–nad5–nad6–nad4L*. Bootstrap values (> 80%) from 1,000 replicates are shown at major nodes. Tip labels correspond to individual sample identifiers. The tree is drawn to scale, with branch lengths in the same units as those of the evolutionary distances to infer the phylogenomic tree. The evolutionary distances were computed using the Maximum Composite Likelihood Method and are in the units of the number of base substitutions per site. The analysis encompassed 168 sequences, representing 10,247 positions. Analysis was conducted using MEGA12. Clades A–D were defined based on phylogenetic clustering. Colour indicates clade assignment as follows: blue (**A**), orange (**B**), green (**C**), and pink (**D**). The tree is rooted using the outgroup *Toxascaris leonina* MN329693 (scale bar: 0.02, Optimal tree with the sum of branch length = 0.308). To the right of the tree, two annotation columns indicate the continent (inner bar) and host species (outer bar) associated with each sample. Continent (Africa (red), Asia (orange), Europe (green), Unknown (grey)) and host (Human (blue), Pig (cyan), Sheep (purple), Non-human Primate (navy), Unknown (dark grey)) are colour-coded as shown in the legend.

To investigate the genetic diversity and genetic clustering of *Ascaris* isolates, we constructed neighbour joining trees based on (i) concatenated nucleotide sequences of all mitochondrial protein-coding genes (Fig. 2), (ii) concatenated amino acid sequences (Supplementary Figure S2) and (iii) each of the 12 individual protein-coding genes (Supplementary Figures S3-14). Across all analyses, consistent clustering patterns were observed, revealing the presence of four well-supported mitochondrial clades, designated A, B, C based on previous assignment^34^, and a newly identified clade D, as well as some potential sub-clades within both clades A and B. Consistent with previous studies^29,34^, clade A comprised the majority of human-derived *Ascaris* samples, forming a large, well-supported cluster with high bootstrap values (100%). Clade B represented a more mixed host population, including both human and non-human hosts, with both clades A and B containing geographically mixed samples.

We identified further samples which were assigned to Clade C, previously identified as comprising *Ascaris* samples from pigs in Europe, from our pool of *Ascaris* samples from Germany. However, the human infection case (DE_H1) which was associated with close contact with pigs via backyard farming practices, along with a subset of pig-derived worms (*n* = 3) from a German abattoir, clustered within Clade A, rather than Clade C.

The novel clade D presented as a genetically distinct and well-supported group, suggesting the presence of a divergent lineage or cryptic population. To our knowledge this is the first time this clade has been described, where all samples within this clade were derived from human hosts in Ethiopia, collected as part of a community-wide deworming initiative^70^. A small number of the samples from Ethiopia (*n* = 10, 16.4%) were assigned to Clades A (*n* = 8) and B (*n* = 2), indicating the presence of multiple mitochondrial lineages circulating within a single geographical setting.

To assess whether the observed clustering pattern was dependent on the tree-building approach, a maximum-likelihood analysis was performed. This broadly retained the clustering observed in the neighbour-joining tree and recovered clade D as a distinct and coherent lineage. While clades B and C were less clearly resolved, the consistent recovery of clade D across analytical approaches supports its designation as a novel mitochondrial clade (Supplementary Figure S15).

We observed strong concordance between phylogenetic placements based on concatenated nucleotide sequences (Fig. 2) and those based on translated amino acid alignments (Supplementary Figure S2). Most samples retained consistent positions across both trees, with only minor within-clade rearrangements. The major clades (A–D) remained well-supported and topologically stable between datasets, indicating that both nucleotide and amino acid data capture similar evolutionary signals. The only sample where this did not apply, was sample HQ704901 (pig, China)^71^ which shifted between clades B and A in the nucleotide and amino acid trees, respectively. However, this placement was not strongly supported in the amino acid analysis and may reflect limited phylogenetic signal or potential technical factors associated with the underlying data. As such, this observation should be interpreted with caution.

**Fig. 3:**
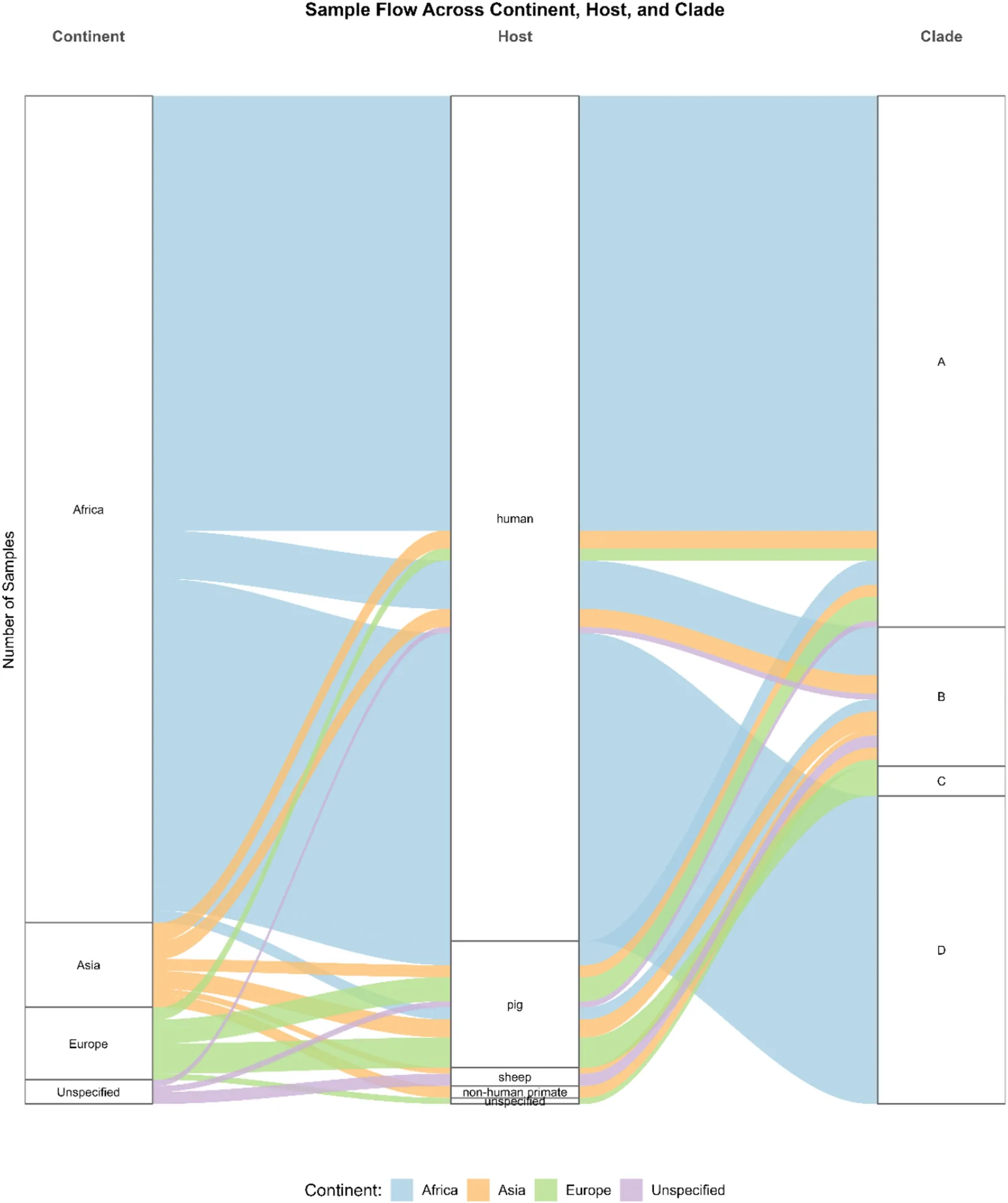
*Alluvial plot illustrating the flow of mitochondrial Ascaris samples across continent of origin*,* host species*,* and mitochondrial clade (collapsed to ****A–D****)*. Stratum widths and flow thicknesses are proportional to the number of samples within each category. The majority of samples originated from humans in Africa and were assigned to clades A or D. Colour represents continent of origin. The plot highlights distinct geographical and host-specific structuring across clades.

Most samples analysed were derived from African populations, predominantly from human hosts, leading to an overrepresentation of African *Ascaris* isolates in the dataset. Despite broad geographical distribution, mitochondrial clades were not uniquely separated by continent or host; however some structuring was evident. In particular, clade C, which contains primarily samples obtained from pigs from Europe (Fig. 3).

### Gene‑wise and concatenated mitochondrial analyses of phylogenetic signal and diversity

**Fig. 4:**
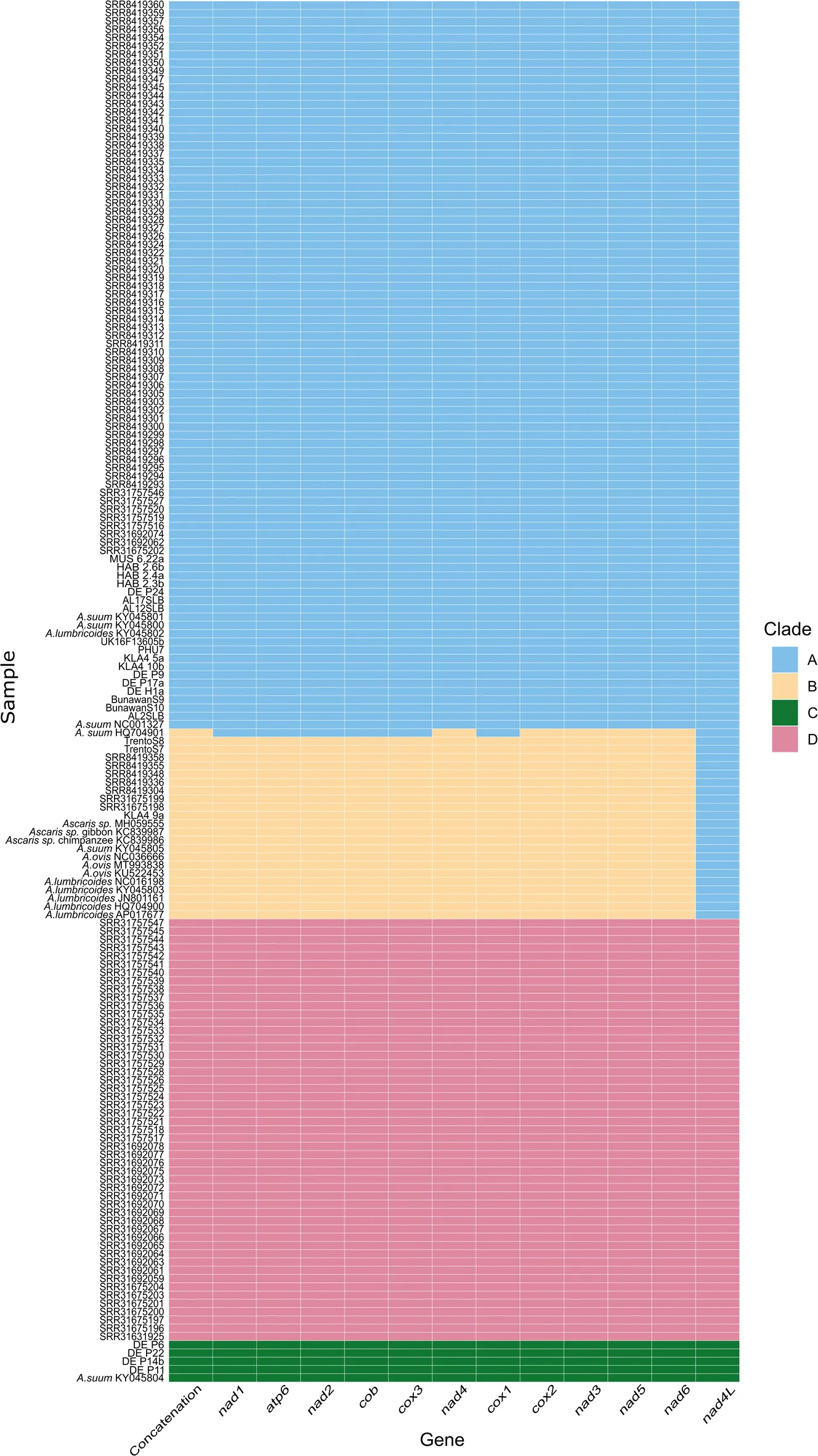
*Heatmap of clade assignment for each mitochondrial gene across all Ascaris samples*. Each row represents a sample, and each column corresponds to a mitochondrial gene, including both individual protein-coding genes and the full concatenated sequence. Colours denote clade assignment per gene, as inferred from phylogenetic analysis. Blue, orange, pink, and green represent clades** A**,** B**,** D**, and** C**, respectively. This visualisation highlights the consistency (or discordance) of clade assignment across genes and samples. Most samples show concordant assignment across all genes, while a minority exhibit inter-gene variability, reflecting gene-specific phylogenetic signal or possible recombination.

To further evaluate whether any single mitochondrial gene contributed disproportionately to the phylogenetic positions, we generated neighbourhood-joining trees for each individual coding gene (Supplementary Figures S2-13), using these to assess concordance across loci rather than to infer gene-specific phylogenies. For each gene, clade membership was determined per sample based on tree visualisation and bootstrap support. The resulting clade associations were compiled into a summary heatmap (Fig. 4), which was largely concordant across individual genes and consistent with the concatenated datasets, indicating that no single gene drives the overall phylogenetic signal. An exception was observed in the gene *nad4L*, a relatively short (~ 234 bp in length^71^, highly conserved gene (Table 2), which clustered clades A and B together, likely reflecting its limited number of phylogenetically informative sites and thus reduced resolution. Sample HQ704901 again showed inconsistent assignment across genes, with 7 genes supporting clade A and 5 supporting clade B.

To evaluate the performance of the assemblies for downstream population genetic analyses, we examined genetic diversity across the mitochondrial genome. All mitogenome assemblies were used to estimate genetic diversity indices across 12 protein-coding genes and the concatenated dataset (Table 2). The number of segregating sites per kb ranged from 95.9 (*cox2*) to 152.8 (*nad4*), demonstrating reliable recovery of variation across genes of varying size and both haplotype and nucleotide diversity (π) values were consistently high. Additionally, estimates of nucleotide diversity (π) and Watterson’s estimator of the population mutation rate (θ), calculated from the number of segregating sites (S) (θW), were broadly concordant, indicating unbiased detection of variation. The *nad4*, *nad5* and *nad6* genes exhibited the highest nucleotide diversity, with *nad4* and *nad5* displaying the greatest average number of pairwise differences (K). The concatenated dataset captured over 1,200 segregating sites and 94 haplotypes, confirming that the reconstructed assemblies retain sufficient resolution to recover population-level diversity across the mitogenome.

**Table 2:** Mitochondrial genetic diversity across reconstructed assemblies.

Gene/Region	Length (bp)*	Segregating sites (S)	S/kb ((S/ Length (bp)*1000)	Haplotypes (h)	Haplotype Diversity (± SD)	π (± SD)	θW	K
Concatenated dataset	10,240	1261	123.2	94	0.987 (0.003)	0.0224 (0.0010)	0.0216	228.9
*nad1*	872	130	149.1	44	0.938 (0.009)	0.0239 (0.0010)	0.0262	20.795
*atp6*	600	75	125.0	44	0.897 (0.015)	0.0177 (0.0009)	0.022	10.594
*nad2*	843	108	128.1	43	0.862 (0.020)	0.0218 (0.0011)	0.0225	18.384
*cob*	1096	141	128.7	41	0.912 (0.013)	0.0202 (0.0011)	0.0226	22.151
*cox3*	777	97	124.8	44	0.883 (0.016)	0.0219 (0.0011)	0.0219	17.009
*nad4*	1230	188	152.8	55	0.954 (0.007)	0.0258 (0.0013)	0.0269	31.785
*cox1*	1534	151	98.4	69	0.966 (0.007)	0.0208 (0.0008)	0.0173	31.224
*cox2*	699	67	95.9	33	0.839 (0.019)	0.0171 (0.0009)	0.0168	11.979
*nad3*	336	34	101.2	21	0.814 (0.020)	0.0232 (0.0010)	0.0178	7.78
*nad5*	1584	188	118.7	61	0.963 (0.007)	0.0257 (0.0011)	0.0209	40.635
*nad6*	435	54	124.1	29	0.814 (0.021)	0.0275 (0.0012)	0.0218	11.967
*nad4L*	234	28	119.7	19	0.801 (0.018)	0.0197 (0.0014)	0.021	4.608

* Excluding gaps and missing data.

Abbreviations: S = number of segregating sites; h = number of haplotypes; π = nucleotide diversity per site; θW = Watterson’s estimator of θ (estimation the population mutation rate (θ), based on the number of segregating sites (S) in a sample); K = average number of pairwise nucleotide differences; SD = standard deviation.

## Discussion

The harnessing of genomic data is becoming increasingly central to public health surveillance, via the monitoring of parasite transmission, the discovery of hotspots of focal infection and identification of zoonotic or geographically driven transmission routes. In this study, we aimed to enhance the assembly of *Ascaris* mitogenomes from low-coverage whole genome sequence data. Although some minor manual curation was still required, the majority of assemblies were developed using freely available, previously validated bioinformatic tools, supporting reproducibility and long-term sustainability.

The removal of host DNA bioinformatically, otherwise known as host sequence depletion is becoming more common in genomic analyses^72,73^. Across the host sequence depletion and mtDNA filtering methods tested in this study, mean complete contig lengths were highly consistent across methods (13.395–13.406 kb), closely matching the expected size of the *Ascaris* mitochondrial genome when measured across the standardised trnN–trnS2 interval (13.381 kb). These values are lower than the full circular mitochondrial genome lengths reported previously (~ 14.1–14.3 kb^28,34,71,74,75^, as the present analysis excludes the highly variable OH/control region.

Complete mitogenome assemblies were recovered for > 99% of samples (*n* = 148/149), with only a single sample requiring substantial manual intervention to resolve. Host sequence depletion methods produced the most reliable assemblies, with Deacon performing best overall^49^. Deacon is a recently developed tool that offers fast performance, particularly on lower-powered computational systems, making it especially well-suited for large-scale analyses and resource-limited environments. Nevertheless, both unfiltered data and mtDNA enrichment approaches performed well, each recovering complete assemblies for > 95% (*n* = 142 for both) of samples.

Previous efforts to reconstruct *Ascaris* mitogenomes from whole genome sequences have relied on reference-guided strategies (i.e. isolating the mtDNA reads through aligning them to a reference, or closely related species), often requiring error correction and the use of GapFiller to span fragmented scaffolds^29^. However, reference-guided approaches risk masking novel variation and may obscure population structure at a local and international level^42,76^, a limitation compounded in *Ascaris* by the limited availability of high-quality reference genomes representing both human- and porcine-derived populations globally^77^. Alternative approaches for mitochondrial genome reconstruction include long-range PCR and hybridisation capture to enrich *Ascaris* mtDNA^41^. Both approaches can improve mitochondrial genome recovery but have distinct limitations. PCR-based methods are prone to amplification bias and may fail to capture the full extent of genetic diversity. Hybridisation capture offers a key advantage in enabling mitochondrial genome recovery from low-parasite-content samples such as eggs in faecal material, where direct whole-genome sequencing not be feasible. However, it is comparatively costly and requires additional laboratory optimisation^41^. *De novo* assembly approaches can mitigate some of these limitations but remain subject to technical challenges, including potential assembly errors and variability associated with data quality and sequencing depth. In this study, we sought to minimise such variability through standardised subsampling and consistent bioinformatic processing. However, direct comparison with alternative approaches (e.g. reference-based mapping or long-range PCR) would be valuable in future work. Using our *Ascaris* mitogenome *de novo* assemblies, we inferred phylogenies from both individual gene trees and concatenated datasets, revealing highly similar topologies, with clade C consistently distinct from the more closely related clades A and B, aligning with previous findings^31–33,78,79^.

In the present dataset, single genes often recovered the same major clades as the concatenated dataset. For *Ascaris*, this supports the continued use of *cox1* for broad mitochondrial lineage assignment and comparison with existing datasets, particularly where complete mitogenome sequencing is not feasible. However, we found that single markers alone may miss signals of introgression or hybridisation and fail to resolve finer-scale structure. For example, sample HQ704901 showed variable placement across genes, clustering with different clades depending on the locus considered. This variability, which has been reported previously^34^, likely reflects limited phylogenetic signal at the level of individual genes, potential technical factors associated with the underlying data or this pattern could also be consistent with more complex biological scenarios (e.g. historical admixture or introgression), which would require confirmation using nuclear genomic data.

A notable finding was the identification of a new clade D, which appears as an intermediate between clade C and the clades A and B, suggesting it may represent a transitional lineage, historical gene flow or incomplete lineage sorting. These samples were obtained from a single community in Ethiopia, collected as part of a longitudinal epidemiological study, where genomic analyses previously demonstrated fine-scale population structure with localised transmission predominantly at the household level rather than random community-wide mixing^70^. Despite an overall reduction in worm populations, likely resulting from mass drug administration efforts, a minority of human hosts continued to harbour disproportionately high worm burdens, which may explain the presence of a small number of worms in clades A and B, where differences in treatment coverage and compliance, multiple introduction events, and household-level micro-environmental factors may allow these ancestral lineages to persist^70^.

In agreement with previous studies, we observed the same clade separation between worm populations infecting humans and those infecting pigs^25,31,80^(Fig. 4). For example, we further identified European samples, in our case from German porcine hosts, in Cluster C. Geographical structuring was evident in our dataset; however, we do note that there is a significant bias towards samples derived from humans in Africa. Previous studies have demonstrated that mitochondrial DNA differentiates between both geographic and species-level variation, as shown in Tanzanian^81^ and Chinese^75^*Ascaris* populations. However, the whole genome sequence of Kenyan isolates indicated extensive interbreeding and a shared nuclear gene pool, despite divergent mitochondrial lineages^29^ and nuclear microsatellite data from the same Tanzanian population suggested high gene flow and limited geographic structure^81^. Data from microsatellite analysis have also been interpreted to support either a single host shift followed by geographical separation^31^ and initial geographical separation followed by multiple host shifts^38^. As microsatellites capture more recent evolutionary events compared to mtDNA, these contrasting signals may be clarified with broader sampling and increased availability of whole genome sequence data across hosts and regions. There are limitations in the use of mitogenomes for phylogenetic studies including the representation of a single maternally inherited locus within mitogenomes, the absence of recombination and incomplete lineage sorting of mitogenomes which may contribute to the discordant phylogenetic patterns observed and means they cannot be used as a substitute for nuclear markers. However, their ability to identify maternal lineages and capture deeper evolutionary events ensures they remain valuable for detecting broad-scale host- and geography-associated patterns.

This study included all publicly available genomic data identified at the time of analysis, as well as newly-generated data, but these datasets were restricted to Africa, Asia and Europe and were unevenly distributed across countries and host groups. Consequently, the distributions reported here should not be considered globally representative or complete. Further global sampling, particularly from underrepresented regions including the Americas and Oceania, is required to identify additional mitochondrial diversity or extend the known distributions of the lineages described.

In conclusion, we present a scalable and reproducible approach for the efficient recovery of high-quality mitogenomes from low-coverage whole-genome sequencing datasets, using standardised, freely available bioinformatic tools, thereby reducing reliance on targeted sequencing approaches. By including host-read depletion and optional mtDNA enrichment prior to *de novo* assembly, we consistently recovered near-complete mitochondrial genomes for the vast majority of samples, demonstrating robustness in the face of variation in input data and analytical method. As a genome skimming approach, this method provides a cost-effective means of generating complete mitochondrial genomes from existing or low-coverage sequencing data, without the need for additional targeted laboratory protocols. While mitochondrial genomes alone cannot resolve introgression or species boundaries between *A. lumbricoides* and *A. suum*, this framework enables scalable generation of mitochondrial datasets that complement nuclear analyses. It facilitated the identification of a novel mtDNA clade and enables broader investigation of *Ascaris* diversity across hosts and regions. By supporting efficient, large-scale data generation, it provides a foundation for expanding genomic resources and advancing studies of parasite diversity, population structure, and transmission.

## Supplementary Information

Below is the link to the electronic supplementary material.

Supplementary Material 1

Supplementary Material 2

## Data Availability

All code is available at: https://github.com/LWoolfe/Ascaris_Mitogenome_Project.git. This includes all bioinformatic tools and R packages. The mtDNA assemblies have been deposited in the European Nucleotide Archive (ENA) as Bioproject PRJEB105081, with assembly accession numbers available via the Github page. The assemblies and the alignments of the 12 individual protein-coding genes and the concatenated genes and corresponding amino acid sequences have been made available via Figshare under the DOI: [https://doi.org/10.6084/m9.figshare.30871202].
