# Supplementary material for: Scalable assembly of Ascaris mitogenomes from whole-genome data reveals a novel clade
Source: Sci Rep. 2026 Sep 11;16:28468. doi: 10.1038/s41598-026-65562-w (PMC13569855; doi:10.1038/s41598-026-65562-w)
Supplement: Supplementary file 1 [file 41598_2026_65562_MOESM2_ESM.docx]

**Supplementary Table S1: Sample Information**

| Country | Host | Sample | Named species | Sequence data source | Reference for sample/data |
| --- | --- | --- | --- | --- | --- |
| China | Human | **HQ704900** | *A. lumbricoides* | Downloaded from NCBI Genome | [1] |
| China | Pig | **HQ704901** | *A.suum* | Downloaded from NCBI Genome | [1] |
| China | Sheep | **MT993838** | *A. ovis* | Downloaded from NCBI Genome | [2] |
| China | Non-Human Primate | **KC839986** | *Ascaris* | Downloaded from NCBI Genome | [3] |
| China | Non-Human Primate | **KC839987** | *Ascaris* | Downloaded from NCBI Genome | [3] |
| Denmark | Unspecified | **MH059555** | *Ascaris* | Downloaded from NCBI Genome | [4] |
| Ethiopia | Human | **SRR31631925 -SRR31675204;**  **SRR31692059;**  **SRR31692061 - SRR31692078; SRR31757516 - SRR31757547** | *A. lumbricoides* | Downloaded from SRA | [5] |
| Germany | Human | **DE_H1a** | *Ascaris* | This study | This study |
| Germany | Pig | **DE_P11** | *Ascaris* | This study | This study |
| Germany | Pig | **DE_P14b** | *Ascaris* | This study | This study |
| Germany | Pig | **DE_P17a** | *Ascaris* | This study | This study |
| Germany | Pig | **DE_P22** | *Ascaris* | This study | This study |
| Germany | Pig | **DE_P24** | *Ascaris* | This study | This study |
| Germany | Pig | **DE_P6** | *Ascaris* | This study | This study |
| Germany | Pig | **DE_P9** | *Ascaris* | This study | This study |
| Hungary | Pig | **PHU7** |  | This study | [6] |
| Kenya | Human | **SRR8419293 -** **SRR8419324;**  **SRR8419324;**  **SRR8419326 - SRR8419336;**  **SRR8419338 - SRR8419345;**  **SRR8419347 - SRR8419360** | *A. lumbricoides* | Downloaded from SRA | [7] |
| Korea | Human | **JN801161** | *A. lumbricoides* | Downloaded from NCBI Genome | [8] |
| Korea | Human | **NC016198** | *A. lumbricoides* | Downloaded from NCBI Genome | [8] |
| Philippines | Pig | **Bunawan S9** | *Ascaris* | This study | [6] |
| Philippines | Pig | **Bunawan S10** | *Ascaris* | This study | [6] |
| Philippines | Pig | **TrentoS7** | *Ascaris* | This study | [6] |
| Philippines | Pig | **TrentoS8** | *Ascaris* | This study | [6] |
| Tanzania | Pig | **KY045800** | *A.suum* | Downloaded from NCBI Genome | [9] |
| Tanzania | Human | **KY045802** | *A. lumbricoides* | Downloaded from NCBI Genome | [9] |
| Tanzania | Human | **KY045803** | *A. lumbricoides* | Downloaded from NCBI Genome | [9] |
| Thailand | Human | **AL12SLB** | *Ascaris* | This study | [10] |
| Thailand | Human | **AL17SLB** | *Ascaris* | This study | [10] |
| Thailand | Human | **AL2SLB** | *Ascaris* | This study | [10] |
| Uganda | Human | **HAB_2_3b** | *Ascaris* | This study | [11] |
| Uganda | Human | **HAB_2_4a** | *Ascaris* | This study | [11] |
| Uganda | Human | **HAB_2_6b** | *Ascaris* | This study | [11] |
| Uganda | Pig | **KLA4_10b** | *Ascaris* | This study | [12] |
| Uganda | Pig | **KLA4_5a** | *Ascaris* | This study | [12] |
| Uganda | Pig | **KLA4_9a** | *Ascaris* | This study | [12] |
| Uganda | Pig | **KY045801** | *A.suum* | Downloaded from NCBI Genome | [9] |
| Uganda | Pig | **KY045805** | *A.suum* | Downloaded from NCBI Genome | [9] |
| Uganda | Human | **MUS_6_22a** | *Ascaris* | This study | [11] |
| United Kingdom | Pig | **KY045804** | *A.suum* | Downloaded from NCBI Genome | [9] |
| United Kingdom | Human | **UK16F13605b** | *Ascaris* | This study | [12] |
| Unspecified | Human | **AP017677** | *A. lumbricoides* | Downloaded from NCBI Genome | Unpublished |
| Unspecified | Sheep | **KU522453** | *A. ovis* | Downloaded from NCBI Genome | Unpublished |
| Unspecified | Pig | **NC001327** | *A. suum* | Downloaded from NCBI Genome | [13] |
| Unspecified | Sheep | **NC036666** | *A. ovis* | Downloaded from NCBI Genome | Unpublished |
| China | Dog | **MN329693** | *Toxascaris leonina* | Downloaded from NCBI Genome | [14] |

References:

1.	Liu, G.-H. *et al.* Comparative analyses of the complete mitochondrial genomes of *Ascaris* *lumbricoides* and *Ascaris* *suum* from humans and pigs. *Gene* **492**, 110–116 (2012).

2.	Chen, Y. *et al.* The mitochondrial genome of the sheep roundworm *Ascaris ovis* (Ascaridida: Nematoda) from Southwest China. *Mitochondrial DNA Part B* **6**, 410–412 (2021).

3.	Xie, Y. *et al.* Complete Mitochondrial Genomes of Chimpanzee- and Gibbon-Derived *Ascaris*Isolated from a Zoological Garden in Southwest China. *PLoS One* **8**, e82795 (2013).

4.	Tams, K. W. *et al.* Parasitic infections and resource economy of Danish Iron Age settlement through ancient DNA sequencing. *PLoS One* **13**, e0197399 (2018).

5.	Landeryou, T. *et al.* Molecular epidemiology of *Ascaris lumbricoides* following multiple rounds of community-wide treatment. *Nat Commun* **16**, 4321 (2025).

6.	Jones, B. Disentangling the role of *Ascaris* β-tubulin isotypes in the emergence of anthelmintic resistance. (University of Surrey, UK, 2023).

7.	Easton, A. *et al.* Molecular evidence of hybridization between pig and human *Ascaris* indicates an interbred species complex infecting humans. *Elife* **9**, 1–26 (2020).

8.	Park, Y. C., Kim, W. & Park, J.-K. The complete mitochondrial genome of human parasitic roundworm, *Ascaris lumbricoides*. *Mitochondrial DNA* **22**, 91–93 (2011).

9.	Nejsum, P. *et al.* *Ascaris* phylogeny based on multiple whole mtDNA genomes. *Infection, Genetics and Evolution* **48**, 4–9 (2017).

10.	Phuphisut, O. *et al.* Transcriptome profiling of male and female *Ascaris lumbricoides* reproductive tissues. *Parasit Vectors* **15**, 477 (2022).

11.	Betson, M. *et al.* Genetic diversity of *Ascaris* in southwestern Uganda. *Trans R Soc Trop Med Hyg* **106**, 75–83 (2012).

12.	Betson, M., Nejsum, P., Bendall, R. P., Deb, R. M. & Stothard, J. R. Molecular epidemiology of ascariasis: A global perspective on the transmission dynamics of *Ascaris* in people and pigs. *J Infect Dis* **210**, 932–941 (2014).

13.	Wolstenholme, D. R., Okimoto, R. & Macfarlane, J. L. Nucleotide correlations that suggest tertiary interactions in the TV-replacement loop-containing mitochondrial tRNAs of the nematodes, *Caenorhabditis elegans* and *Ascaris suum*. *Nucleic Acids Res* **22**, 4300–4306 (1994).

14.	Xie, Y. *et al.* Characterization of the complete mitochondrial genome sequence of the dog roundworm *Toxascaris leonina* (Nematoda, Ascarididae) from China. *Mitochondrial DNA Part B* **4**, 3517–3519 (2019).

**Supplementary Table S2: mtDNA Assembly decontamination etc. results (excel file)**

**Supplementary Table S3: Summary of mapping metrics for *De Novo* assembled mitochondrial genomes**. Analysis of subsampled sequencing reads mapped back to their respective assemblies using Bowtie2 *successfully covering the mitochondrial genome

| Sample ID | Length | Number of reads* | Coverage | Mean depth per base | Mean base quality | Mean mapping quality |
| --- | --- | --- | --- | --- | --- | --- |
| AL12SLB | 14107 | 2664836 | 99.84 | 28322.1 | 35.4 | 41.7 |
| AL17SLB | 14314 | 3805662 | 100 | 39858.1 | 35.6 | 41.7 |
| AL2SLB | 14266 | 1793415 | 100 | 18844.3 | 35.4 | 41.7 |
| Bunawan S10 | 14300 | 5344565 | 100 | 54755.6 | 35.1 | 41.6 |
| Bunawan S9 | 14484 | 772984 | 100 | 7741.83 | 35 | 41.4 |
| DE H1a | 14429 | 1298423 | 99.99 | 13465 | 39.5 | 41.6 |
| DE P11 | 14089 | 3596447 | 100 | 37962.4 | 39.5 | 41.8 |
| DE P14b | 14027 | 8896601 | 100 | 94984.1 | 39.5 | 41.8 |
| DE P17a | 13833 | 3810451 | 100 | 40958 | 39.4 | 41.7 |
| DE P22 | 14014 | 6133917 | 100 | 65531.1 | 39.5 | 41.8 |
| DE P24 | 13967 | 8390230 | 100 | 89825.7 | 39.5 | 41.8 |
| DE P6 | 14082 | 4837586 | 100 | 51326.7 | 39.5 | 41.8 |
| DE P9 | 14418 | 6110136 | 100 | 63401.8 | 39.5 | 41.7 |
| HAB 2.3b | 14300 | 5612739 | 100 | 58197.3 | 35.6 | 41.6 |
| HAB 2.4a | 13882 | 5389733 | 100 | 57195.5 | 35.6 | 41.6 |
| HAB 2.6b | 13968 | 1676488 | 100 | 17803.3 | 35.6 | 41.7 |
| KLA 4.10b | 14236 | 6550649 | 100 | 68626.4 | 35.6 | 41.6 |
| KLA 4.5a | 13972 | 4356068 | 100 | 46624.1 | 35.8 | 41.7 |
| KLA 4.9a | 14299 | 15101947 | 100 | 158022 | 35.8 | 41.8 |
| MUS 6.22a | 13895 | 1606977 | 100 | 17125.8 | 35.7 | 41.6 |
| PHU7 | 13943 | 2346481 | 100 | 25228 | 35.4 | 41.5 |
| Trento S7 | 14384 | 379796 | 100 | 3651.48 | 35.1 | 41.4 |
| Trento S8 | 14282 | 108584 | 100 | 937.03 | 35.2 | 41.4 |
| SRR31675200 | 14063 | 1974429 | 100 | 21053.6 | 35.6 | 41.7 |
| SRR31675201 | 14495 | 1456871 | 100 | 15070.9 | 35.6 | 41.7 |
| SRR31675202 | 14338 | 1646436 | 100 | 17219.3 | 34.8 | 41.4 |
| SRR8419293 | 14322 | 14078167 | 100 | 92275.4 | 37 | 41.7 |
| SRR8419294 | 14431 | 5195710 | 100 | 33609.6 | 36.5 | 41.5 |
| SRR8419295 | 13942 | 7420389 | 100 | 49482.6 | 36.4 | 41.6 |

**Supplementary Figure S1**. Assembly of the mitochondrial genome of *Ascaris* spp. generated using MITOZ with annotation from MITOS2. The edited mitochondrial genome was linearised for visualisation, and ordered to start at *nad1* and displays the relative positions and orientations of annotated genes. Protein-coding genes are shown in red, transfer RNA (tRNA) genes in blue, and ribosomal RNA (rRNA) genes in green. The grey region at the right end (labelled “OH”) represents the putative AT-rich control region, corresponding to the origin of replication for the heavy strand. Gene annotations and boundaries were predicted using the invertebrate mitochondrial genetic code. Direction of arrows indicates the transcriptional orientation of each gene

**Supplementary Figure S2**: *Neighbour-joining tree of concatenated mitochondrial amino acid sequences from* Ascaris *samples.* The tree was constructed using concatenated amino acid sequences from 12 mitochondrial protein-coding genes in the order: *nad1–atp6–nad2–cob–cox3–nad4–cox1–cox2–nad3–nad5–nad6–nad4L*. Bootstrap values from 1,000 replicates are shown at major nodes. Evolutionary distances are inferred using the Poisson correction method and are in the unit of amino acid substitution per site. The pairwise deletion option was applied to all the ambiguous positions for each sequence resulting in a final dataset comprising of 3,405 positions. Analysis was conducted using MEGA12. Samples were grouped into major clades (A–D) based on phylogenetic topology and bootstrap support. Colour indicates clade assignment as follows: blue (A), orange (B), green (C), and pink (D). The tree is rooted using the outgroup *Toxascaris leonina* MN329693 (scale bar: 0.02 nucleic acid substitutions per site). To the right of the tree, two annotation columns indicate the continent (inner bar) and host species (outer bar) associated with each sample. Continent (Africa (red), Asia (orange), Europe (green), Unknown (grey)) and host (Human (blue), Pig (cyan), Sheep (purple), Non-human Primate (navy), Unknown (dark grey)) are colour-coded as shown in the legend.

**Supplementary Figure S3: *Neighbour-joining tree of cox1 from* *Ascaris* *samples****.*
Bootstrap values (>80%) from 1,000 replicates are shown at internal nodes. Tip labels correspond to individual sample identifiers. The tree is drawn to scale, with branch lengths in the same units as those of the evolutionary distances to infer the phylogenomic tree. The evolutionary distances were computed using the Maximum Composite Likelihood Method and are in the units of the number of base substitutions per site. The analysis encompassed 168 sequences aligned using MEGA12. Clades A–D were defined based on phylogenetic clustering. Colour indicates clade assignment as follows: blue (A), orange (B), green (C), and pink (D). The tree is rooted using the outgroup *Toxascaris leonina* MN329693 (scale bar: 0.02 nucleotide substitutions per base).

**Supplementary Figure S4 *Neighbour-joining tree of atp6 from* *Ascaris* *samples.***
Bootstrap values (>80%) from 1,000 replicates are shown at internal nodes. Tip labels correspond to individual sample identifiers. The tree is drawn to scale, with branch lengths in the same units as those of the evolutionary distances to infer the phylogenomic tree. The evolutionary distances were computed using the Maximum Composite Likelihood Method and are in the units of the number of base substitutions per site. The analysis encompassed 168 sequences aligned using MEGA12. Clades A–D were defined based on phylogenetic clustering. Colour indicates clade assignment as follows: blue (A), orange (B), green (C), and pink (D). The tree is rooted using the outgroup *Toxascaris leonina* MN329693 (scale bar: 0.02 nucleotide substitutions per base).

**Supplementary Figure S5 *Neighbour-joining tree of cob from* *Ascaris* *samples.***
Bootstrap values (>80%) from 1,000 replicates are shown at internal nodes. Tip labels correspond to individual sample identifiers. The tree is drawn to scale, with branch lengths in the same units as those of the evolutionary distances to infer the phylogenomic tree. The evolutionary distances were computed using the Maximum Composite Likelihood Method and are in the units of the number of base substitutions per site. The analysis encompassed 168 sequences, aligned using MEGA12. Clades A–D were defined based on phylogenetic clustering. Colour indicates clade assignment as follows: blue (A), orange (B), green (C), and pink (D). The tree is rooted using the outgroup *Toxascaris leonina* MN329693 (scale bar: 0.02 nucleotide substitutions per base).

**Supplementary Figure S6 *Neighbour-joining tree of cox2 from* *Ascaris* *samples.***
Bootstrap values (>80%) from 1,000 replicates are shown at internal nodes. Tip labels correspond to individual sample identifiers. The tree is drawn to scale, with branch lengths in the same units as those of the evolutionary distances to infer the phylogenomic tree. The evolutionary distances were computed using the Maximum Composite Likelihood Method and are in the units of the number of base substitutions per site. The analysis encompassed 168 sequences, aligned using MEGA12. Clades A–D were defined based on phylogenetic clustering. Colour indicates clade assignment as follows: blue (A), orange (B), green (C), and pink (D). The tree is rooted using the outgroup *Toxascaris leonina* MN329693 (scale bar: 0.02 nucleotide substitutions per base).

**Supplementary Figure S7 *Neighbour-joining tree of cox3 from* *Ascaris* *samples****.*
Bootstrap values (>80%) from 1,000 replicates are shown at internal nodes. Tip labels correspond to individual sample identifiers. The tree is drawn to scale, with branch lengths in the same units as those of the evolutionary distances to infer the phylogenomic tree. The evolutionary distances were computed using the Maximum Composite Likelihood Method and are in the units of the number of base substitutions per site. The analysis encompassed 168 sequences, aligned using MEGA12. Clades A–D were defined based on phylogenetic clustering. Colour indicates clade assignment as follows: blue (A), orange (B), green (C), and pink (D). The tree is rooted using the outgroup *Toxascaris leonina* MN329693 (scale bar: 0.02 nucleotide substitutions per base).

**Supplementary** **Figure S8 *Neighbour-joining tree of nad1 from* *Ascaris* *samples.***
Bootstrap values (>80%) from 1,000 replicates are shown at internal nodes. Tip labels correspond to individual sample identifiers. The tree is drawn to scale, with branch lengths in the same units as those of the evolutionary distances to infer the phylogenomic tree. The evolutionary distances were computed using the Maximum Composite Likelihood Method and are in the units of the number of base substitutions per site. The analysis encompassed 168 sequences, aligned using MEGA12. Clades A–D were defined based on phylogenetic clustering. Colour indicates clade assignment as follows: blue (A), orange (B), green (C), and pink (D). The tree is rooted using the outgroup *Toxascaris leonina* MN329693 (scale bar: 0.02 nucleotide substitutions per base).

**Supplementary Figure S9 *Neighbour-joining tree of nad2 from* *Ascaris* *samples.***
Bootstrap values (>80%) from 1,000 replicates are shown at internal nodes. Tip labels correspond to individual sample identifiers. The tree is drawn to scale, with branch lengths in the same units as those of the evolutionary distances to infer the phylogenomic tree. The evolutionary distances were computed using the Maximum Composite Likelihood Method and are in the units of the number of base substitutions per site. The analysis encompassed 168 sequences, aligned using MEGA12. Clades A–D were defined based on phylogenetic clustering. Colour indicates clade assignment as follows: blue (A), orange (B), green (C), and pink (D). The tree is rooted using the outgroup *Toxascaris leonina* MN329693 (scale bar: 0.02 nucleotide substitutions per base).

**Supplementary Figure S10 *Neighbour-joining tree of nad3 from* *Ascaris* *samples.***
Bootstrap values (>80%) from 1,000 replicates are shown at internal nodes. Tip labels correspond to individual sample identifiers. The tree is drawn to scale, with branch lengths in the same units as those of the evolutionary distances to infer the phylogenomic tree. The evolutionary distances were computed using the Maximum Composite Likelihood Method and are in the units of the number of base substitutions per site. The analysis encompassed 168 sequences, aligned using MEGA12. Clades A–D were defined based on phylogenetic clustering. Colour indicates clade assignment as follows: blue (A), orange (B), green (C), and pink (D). The tree is rooted using the outgroup *Toxascaris leonina* MN329693 (scale bar: 0.02 nucleotide substitutions per base).

**Supplementary Figure S11 *Neighbour-joining tree of nad4 from* *Ascaris* *samples.***
Bootstrap values (>80%) from 1,000 replicates are shown at internal nodes. Tip labels correspond to individual sample identifiers. The tree is drawn to scale, with branch lengths in the same units as those of the evolutionary distances to infer the phylogenomic tree. The evolutionary distances were computed using the Maximum Composite Likelihood Method and are in the units of the number of base substitutions per site. The analysis encompassed 168 sequences, aligned using MEGA12. Clades A–D were defined based on phylogenetic clustering. Colour indicates clade assignment as follows: blue (A), orange (B), green (C), and pink (D). The tree is rooted using the outgroup *Toxascaris leonina* MN329693 (scale bar: 0.02 nucleotide substitutions per base).

**Supplementary Figure S12 *Neighbour-joining tree of nad4l from* *Ascaris* *samples.***
Bootstrap values (>80%) from 1,000 replicates are shown at internal nodes. Tip labels correspond to individual sample identifiers. The tree is drawn to scale, with branch lengths in the same units as those of the evolutionary distances to infer the phylogenomic tree. The evolutionary distances were computed using the Maximum Composite Likelihood Method and are in the units of the number of base substitutions per site. The analysis encompassed 168 sequences, aligned using MEGA12. Clades A–D were defined based on phylogenetic clustering. Colour indicates clade assignment based on concatenated dataset, which is as follows: blue (A), orange (B), green (C), and pink (D). The tree is rooted using the outgroup *Toxascaris leonina* MN329693 (scale bar: 0.02 nucleotide substitutions per base).

**Supplementary Figure S13 *Neighbour-joining tree of nad5 from* *Ascaris* *samples****.*
Bootstrap values (>80%) from 1,000 replicates are shown at internal nodes. Tip labels correspond to individual sample identifiers. The tree is drawn to scale, with branch lengths in the same units as those of the evolutionary distances to infer the phylogenomic tree. The evolutionary distances were computed using the Maximum Composite Likelihood Method and are in the units of the number of base substitutions per site. The analysis encompassed 168 sequences, aligned using MEGA12. Clades A–D were defined based on phylogenetic clustering. Colour indicates clade assignment as follows: blue (A), orange (B), green (C), and pink (D). The tree is rooted using the outgroup *Toxascaris leonina* MN329693 (scale bar: 0.02 nucleotide substitutions per base).

**Supplementary Figure S14 *Neighbour-joining tree of nad6 from* *Ascaris* *samples.***
Bootstrap values (>80%) from 1,000 replicates are shown at internal nodes. Tip labels correspond to individual sample identifiers. The tree is drawn to scale, with branch lengths in the same units as those of the evolutionary distances to infer the phylogenomic tree. The evolutionary distances were computed using the Maximum Composite Likelihood Method and are in the units of the number of base substitutions per site. The analysis encompassed 168 sequences, aligned using MEGA12. Clades A–D were defined based on phylogenetic clustering. Colour indicates clade assignment as follows: blue (A), orange (B), green (C), and pink (D). The tree is rooted using the outgroup *Toxascaris leonina* MN329693 (scale bar: 0.02 nucleotide substitutions per base).

**Supplementary Figure S15 Maximum-likelihood phylogeny of concatenated mitochondrial protein-coding genes from *Ascaris* spp.**
The tree was inferred using IQ-TREE under the best-fit substitution model selected by ModelFinder. Branch support ≥ 70 values are shown at nodes (ultrafast bootstrap / SH-aLRT). The tree is rooted using *Toxascaris leonina* as an outgroup. Samples are coloured according to clade assignment based on neighbour-joining analysis, highlighting four major groupings (A (blue), B (red), C (green) and D(purple))

**Supplementary Table S5: Accession number of ascarid species used for mtDNA enrichment using Deacon**

| **Species** | **Accession Number(s)** |
| --- | --- |
| *Ascaris lumbricoides* | AP017677.1  HQ704900.1  KY045803.1  SRR31757522  SRR31757533 |
| *Parascaris equorum* | AP017696.1 |
| *Ascaris sp. chimpanzee-cA* | KC839986.1 |
| *Ascaris suum* | NC_001327  HQ704901.1  KY045800.1  KY045804.1 |
| *Baylisascaris procyonis* | NC_016200 |
| *Toxascaris leonina TLST* | MW560284.1 |
